# Remissão de Bloqueio de Ramo Esquerdo na Cardiomiopatia Alcoólica após Tratamento

**DOI:** 10.36660/abc.20250327

**Published:** 2025-11-26

**Authors:** Manuela Cristina Ribeiro Dias Barroso, Heleutério da Conceição Nicolau Madogolele, Deborah de Sá Pereira Belfort, Bruno Biselli, Silvia Moreira Ayub-Ferreira, Edimar Alcides Bocchi

**Affiliations:** 1 Instituto do Coração do Hospital das Clínicas da Faculdade de Medicina da Universidade de São Paulo São Paulo SP Brasil Instituto do Coração do Hospital das Clínicas da Faculdade de Medicina da Universidade de São Paulo, São Paulo, SP – Brasil

**Keywords:** Bloqueio de Ramo, Cardiomiopatia Alcoólica, Remodelação Ventricular

## Introdução

O bloqueio do ramo esquerdo ocorre no contexto de diversas cardiomiopatias; entretanto, sua reversão é raramente descrita. Descrevemos um caso de cardiomiopatia alcoólica com bloqueio do ramo esquerdo que apresentou reversão após instituição de tratamento direcionado e cessação do consumo de álcool.

## Descrição do caso

Um paciente de 38 anos com histórico de abuso de álcool de longa data, iniciado aos 16 anos, com um padrão de consumo de três porções diárias de 350 mL de bebidas destiladas (aproximadamente 370 g/dia de álcool), se apresentou para avaliação.

Aos 36 anos, apresentou seu primeiro episódio de descompensação clínica, compatível com insuficiência cardíaca tipo B, em outra instituição. Durante a internação, o cateterismo cardíaco não revelou lesões coronárias obstrutivas. Durante os dois anos que antecederam sua admissão em nossa clínica, o paciente havia sido acompanhado em um serviço externo com doses subterapêuticas de medicamentos para insuficiência cardíaca, conforme as diretrizes. Devido a um novo início de dispneia, o paciente foi encaminhado ao nosso centro para avaliação.

**Figura 1:**
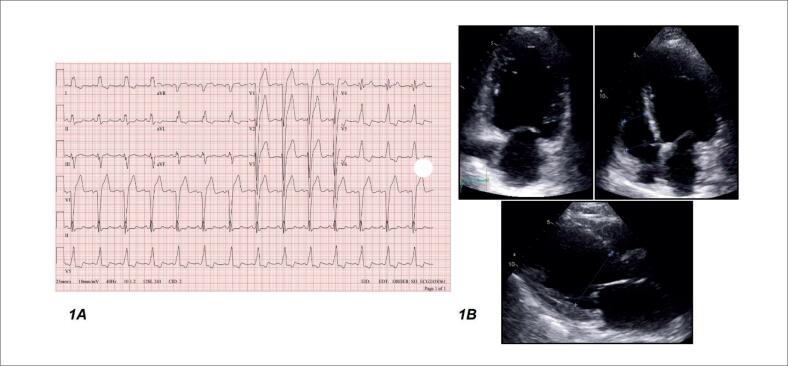
A) Eletrocardiograma mostrando a presença de bloqueio do ramo esquerdo com duração do QRS de 154 milissegundos. B) Ecocardiograma transtorácico mostrando dilatação do átrio esquerdo e ventrículo esquerdo, com fração de ejeção do ventrículo esquerdo de 34%.

O exame de sangue inicial revelou peptídeo natriurético tipo B (BNP) 471 [NR < 100pg/mL], Na 137 (NR 136-145mEq/L), K 4,7 (NR 3,5-5,0 mEq/L), Mg 2,1 (NR 1,7-2,2mg/dl), iCa 1,4 (NR 1,1-1,4 mmol/L). O eletrocardiograma (ECG) mostrou dilatação sinusal e atrial esquerda e a presença de bloqueio de ramo esquerdo com duração do QRS de 154 milissegundos (Figura 1A), também relatado 2 anos antes. O índice cardiotorácico estava aumentado na radiografia de tórax, e a cefalização dos vasos pulmonares sugeria congestão pulmonar.

O ecocardiograma transtorácico demonstrou dilatação significativa do átrio e ventrículo esquerdos (átrio esquerdo (AE) 59 mm [NR: 30-40 mm]; diâmetro diastólico final do ventrículo esquerdo (DDVE) 74 mm [NR: 42-58 mm]; diâmetro sistólico final do ventrículo esquerdo (DSVE) 62 mm [NR: 25-40 mm]), com fração de ejeção do ventrículo esquerdo (FEVE) de 34% e hipocinesia difusa, função ventricular direita moderada, além da presença de regurgitação tricúspide e mitral moderadas (Figura 1B).

Para o diagnóstico diferencial da etiologia da insuficiência cardíaca com fração de ejeção reduzida (ICFEr), foram solicitados os seguintes exames: sorologia para doença de Chagas, monitorização ambulatorial da pressão arterial, sorologia para hepatite e HIV, hemoglobina glicada, testes de função tireoidiana e avaliação da função renal. Todos os resultados estavam dentro dos limites da normalidade. Além disso, foi realizada ressonância magnética cardíaca, que não revelou realce miocárdico tardio com gadolínio, FEVE de 34% e nenhuma evidência de edema miocárdico.

No início do acompanhamento, o regime de tratamento para insuficiência cardíaca do paciente incluía espironolactona 25 mg uma vez ao dia, carvedilol 3,125 mg duas vezes ao dia e enalapril 5 mg duas vezes ao dia; esses agentes foram titulados para as doses máximas toleradas — espironolactona 25 mg uma vez ao dia, enalapril 10 mg duas vezes ao dia e carvedilol 25 mg a cada 12 horas — e dapagliflozina 10 mg uma vez ao dia foi adicionada ao regime. O paciente foi orientado a cessar o consumo de álcool. Após um ano de tratamento e cessação do consumo de álcool, o paciente estava assintomático.

**Figura 2:**
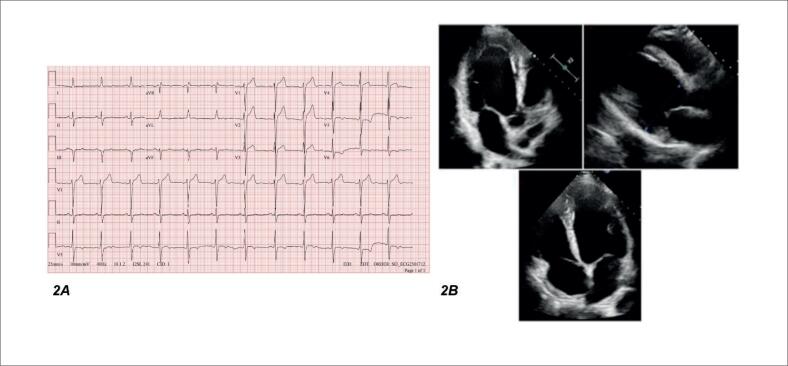
A) Eletrocardiogramaresolução do bloqueio do ramo esquerdo, com duração do QRS de 110 milissegundos. B) Ecocardiograma transtorácico mostrando dilatação discreta do átrio esquerdo e dimensões normais do ventrículo, fração de ejeção do ventrículo esquerdo de 33%.

O ECG de rotina mostrou resolução do bloqueio do ramo esquerdo do ramo esquerdo (BRE), com redução acentuada da duração do QRS (110 vs. 154 ms), menor intervalo PR (154 vs. 174 ms) e normalização do QT corrigido (QTc 419 vs. 491 ms), mantendo o ritmo sinusal, sem sinais indiretos de dilatação do AE (Figura 2A). O Holter mostrou ritmo sinusal com frequência cardíaca mínima de 60 bpm, média de 84 bpm e máxima de 122 bpm. Não foram observadas arritmias ventriculares ou supraventriculares; a condução atrioventricular manteve o intervalo PR dentro dos limites normais e a condução intraventricular não apresentou alterações significativas.

O BNP de acompanhamento foi de 10pg/mL e o ecocardiograma transtorácico demonstrou praticamente normalização das dimensões das cavidades esquerdas (AE 42mm DDVE 55mm DSVE 45mm), redução da regurgitação mitral e tricúspide — de moderada para discreta e leve, respectivamente, melhora da função ventricular direita, progredindo de disfunção moderada para excursão sistólica do plano anular tricúspide (TAPSE) de 10 mm para função normal com TAPSE de 18 mm e FEVE de 33% (Figura 2B).

## Discussão

Nosso caso apresenta um achado raro e digno de nota: a reversão do BRE após a cessação do consumo de álcool e o início da terapia medicamentosa para ICFEr, conforme as diretrizes, em um paciente com cardiomiopatia alcoólica (CMA). Esse fenômeno é raramente relatado na literatura.

O BRE é um modelo único de dessincronia eletromecânica ventricular e é suspeito de gerar remodelação estrutural, disfunção do VE e pior prognóstico.^1^ A prevalência de BRE é <1%, mas aumenta com a idade, até 5% aos 80 anos.^1^ A maioria dos pacientes com BRE apresenta evidências de doença cardíaca estrutural, como doença arterial coronariana, alterações degenerativas, condições infiltrativas e cardiomiopatias, incluindo CMA.^1^

O bloqueio reversível do ramo geralmente se enquadra em sete categorias: (1) bloqueio dependente da frequência ("funcional") que aparece apenas em frequências cardíacas muito rápidas ou muito lentas; (2) isquemia transitória ou espasmo coronário durante infarto agudo do miocárdio, resolvendo-se após reperfusão; (3) distúrbios eletrolíticos graves, especialmente hipercaliemia, nos quais a normalização do K^+^ sérico restaura a condução; (4) fármacos ou toxinas bloqueadores dos canais de sódio (por exemplo, flecainida, lacosamida, antidepressivos tricíclicos, intoxicação por digoxina); (5) edema inflamatório de miocardite de origem viral, autoimune, de Lyme ou inibidor de ponto de verificação; (6) trauma torácico ou edema septal pós-procedimento, que geralmente desaparece em 24–48 horas; e (7) sobrecarga hemodinâmica aguda ou cardiomiopatia reversível na insuficiência cardíaca descompensada, onde a otimização médica e a "remodelação reversa" podem estreitar o QRS.^2,3^

Embora o tratamento farmacológico para insuficiência cardíaca seja bem conhecido por induzir a remodelação reversa e melhorar os desfechos clínicos, a reversão do BRE não é comumente observada. De fato, estudos sugerem que, em pacientes com insuficiência cardíaca e BRE, a terapia de ressincronização cardíaca (TRC) pode ser superior à terapia medicamentosa isolada na melhora da função cardíaca.^4^

Apenas alguns casos na literatura descrevem BRE reversível. Um relato sugeriu melhora do BRE após tratamento com sacubitril/valsartana, embora a causalidade nesse caso seja discutível.^5^ Notavelmente, nossa paciente apresentou resolução do BRE sem o uso de sacubitril/valsartana, destacando a potencial contribuição de outros mecanismos, particularmente a cessação do consumo de álcool e a terapia padrão otimizada para ICFEr.

Fisiopatologicamente, a CMA é caracterizada pelos efeitos tóxicos diretos do etanol e seus metabólitos no miocárdio, promovendo estresse oxidativo, disfunção mitocondrial, inflamação e fibrose. Essas alterações não apenas prejudicam a contratilidade miocárdica, mas também podem afetar o sistema de condução cardíaco.^6^ Estudos experimentais em modelos caninos demonstraram que a exposição crônica ao álcool pode levar a atrasos na condução e alterações estruturais no sistema de condução ventricular, corroborando a hipótese de potencial reversibilidade com a remoção do insulto tóxico.^7^

Além disso, aproximadamente metade a dois terços dos pacientes com CMA não apresentam fibrose miocárdica detectável por realce tardio com gadolínio, um achado que provavelmente reflete um estágio mais precoce da doença ou uma suscetibilidade individual a lesões funcionais sem remodelação estrutural. A ausência de fibrose está associada a uma melhor resposta à cessação do consumo de álcool e, possivelmente, a uma menor mortalidade.^6^

As explicações propostas para a fibrose ausente ou mínima incluem o tempo de exposição e o estágio da doença — a disfunção miocárdica na CMA pode surgir antes da deposição da matriz extracelular, especialmente em bebedores mais jovens ou aqueles com menor duração do abuso; toxicidade predominantemente funcional — alterações metabólicas (estresse oxidativo, disfunção mitocondrial) e inflamatórias podem deprimir a contratilidade sem causar necrose extensa; e fatores moduladores, como níveis de zinco, polimorfismos em enzimas metabolizadoras de etanol e cardiomiocinas como FGF-21, que foram implicados em modelos experimentais como protetores contra a fibrogênese.^8^

Assim, em nosso caso, a reversão do BRE pode ser explicada por uma combinação de recuperação miocárdica estrutural e funcional, impulsionada tanto pela abstinência de álcool quanto pela terapia medicamentosa otimizada. Esse achado ressalta a importância da cessação do consumo de álcool no tratamento da CMA e contribui para a limitada literatura, documentando um caso raro de recuperação eletrocardiográfica em um cenário clínico claramente definido.

As limitações clássicas da singularidade e, portanto, da generalização limitada incluem a ausência de um grupo de controle ou séries comparativas que impedem o estabelecimento de um nexo causal firme entre a resolução do bloqueio do ramo esquerdo e a combinação de abstinência alcoólica com titulação farmacológica; o curto acompanhamento e a escassez de ECGs seriais ou monitoramento contínuo não permitem que a cronologia exata seja definida ou que a durabilidade da normalização do QRS seja garantida; e vários medicamentos foram titulados simultaneamente, obscurecendo o efeito isolado de cada um.

## Conclusão

Em resumo, este caso ilustra uma reversão excepcionalmente rara de BRE em um paciente com cardiomiopatia alcoólica, provavelmente motivado por terapia médica otimizada orientada por diretrizes e cessação sustentada do consumo de álcool, levando à remodelação cardíaca favorável.

## Data Availability

Os conteúdos subjacentes ao texto da pesquisa estão contidos no manuscrito.
