# Supplementary material for: Remissão de Bloqueio de Ramo Esquerdo na Cardiomiopatia Alcoólica após Tratamento
Source: Arq Bras Cardiol. 2025 Nov 26;122(10):e20250327. [Article in Portuguese] doi: 10.36660/abc.20250327 (PMC12671594; doi:10.36660/abc.20250327)
Supplement: Translation

# Translation: Reversal of Left Bundle Branch Block Following Treatment in Alcoholic Cardiomyopathy

**DOI:** 10.36660/abc.20250327i

**Authors:** Manuela Cristina Ribeiro Dias Barroso, Heleutério da Conceição Nicolau Madogolele, Deborah de Sá Pereira Belfort, Bruno Biselli, Silvia Moreira Ayub-Ferreira, Edimar Alcides Bocchi

**Affiliations:** 1 São Paulo SP Brazil Instituto do Coração do Hospital das Clínicas da Faculdade de Medicina da Universidade de São Paulo, São Paulo, SP – Brazil

**Keywords:** Bundle-Branch Block, Alcoholic Cardiomyopathy, Ventricular Remodeling

## Introduction

Left bundle branch block occurs in the context of several cardiomyopathies; however, its reversal is rarely described. We describe a case of alcoholic cardiomyopathy with left bundle branch block that presented reversal after institution of targeted treatment and alcohol cessation.

## Case description

A 38-year-old patient with a long-standing history of alcohol abuse, commencing at the age of 16, with a consumption pattern of three daily servings of 350 mL of distilled spirits (approximately 370 g/day of alcohol), presented for evaluation.

At the age of 36, he experienced his first episode of clinical decompensation, consistent with profile B heart failure at another institution. During hospitalization, cardiac catheterization revealed no obstructive coronary lesions. During the two years preceding his admission to our clinic, the patient had been followed at an outside facility on subtherapeutic doses of guideline-directed heart-failure medications. Due to a new onset of dyspnea, the patient was referred to our center for assessment.

Initial blood test revealed B-type natriuretic peptide (BNP) 471 [NR < 100pg/mL], Na 137 (NR 136-145mEq/L), K 4,7 (NR 3,5-5,0 mEq/L), Mg 2,1 (NR 1,7-2,2mg/dl), iCa 1,4 (NR 1.1-1.4 mmol/L). Electrocardiogram (ECG) showed sinus, left atrial dilatation, and the presence of left bundle branch block with QRS duration of 154 milliseconds (Figure 1A), which had also been reported 2 years earlier. The cardiothoracic index was increased in the chest x-ray, and cephalization of the pulmonary vessels was suggestive of pulmonary congestion.

**Figure 1:** A) Electrocardiogram showing the presence of left bundle branch block with QRS duration of 154 milliseconds. B) Transthoracic echocardiogram showing dilation of the left atrium and left ventricle, with left ventricular ejection fraction of 34%.

Transthoracic echocardiogram demonstrated significant dilation of the left atrium and ventricle (Left atrium (LA) 59 mm [NR: 30-40mm]; Left ventricular end diastolic diameter (LVEDD) 74 mm [NR: 42-58mm] Left ventricular end systolic diameter (LVESD) 62mm [NR: 25-40mm]), with left ventricular ejection fraction of (LVEF) 34% and diffuse hypokinesia, moderate right-ventricular function, additionally the presence of moderate tricuspid and mitral regurgitation (Figure 1B).

For differential diagnosis of the etiology of heart failure with reduced ejection fraction (HFrEF), the following tests were requested: serology for Chagas disease, ambulatory blood pressure monitoring, serology for hepatitis and HIV, glycated hemoglobin, thyroid function tests, and renal function assessment. All results were within normal limits. Additionally, cardiac magnetic resonance imaging was performed, revealing no late gadolinium myocardial enhancement, LVEF of 34% and no evidence of myocardial edema.

At the start of follow-up, the patient's heart-failure regimen comprised spironolactone 25 mg once daily, carvedilol 3.125 mg twice daily, and enalapril 5 mg twice daily; these agents were uptitrated to the maximally tolerated doses—spironolactone 25 mg once daily, enalapril 10 mg twice daily, and carvedilol 25 mg every 12 hours—and dapagliflozin 10 mg once daily was added to the regimen. The patient was counseled to cease alcohol consumption. After one year of treatment and cessation of alcohol consumption, the patient was asymptomatic.

The routine ECG showed resolution of the LBBB, with marked reduction in QRS duration (110 vs 154 ms), shorter PR interval (154 vs 174 ms), and normalization of the corrected QT (QTc 419 vs 491 ms), maintaining sinus rhythm, without indirect signs of LA dilatation (Figure 2A). Holter monitoring showed sinus rhythm with a minimum heart rate of 60 bpm, an average of 84 bpm, and a maximum of 122 bpm. No ventricular or supraventricular arrhythmias were observed; atrioventricular conduction maintained a PR interval within normal limits, and intraventricular conduction showed no significant changes.

**Figure 2:** A) Electrocardiogram resolution of the left bundle branch block, with QRS duration of 110 milliseconds. B) Transthoracic echocardiogram showing mild left atrium dilatation and normal ventricle dimensions, a left ventricular ejection fraction of 33%.

Follow-up BNP was 10pg/mL and transthoracic echocardiogram demonstrated practically normalization of the dimensions of the left cavities (LA 42mm LVEDD 55mm LVESD 45mm), a reduction in mitral and tricuspid regurgitation—from moderate to trace and mild, respectively, an improvement in right-ventricular function, progressing from moderate dysfunction with a tricuspid annular plane systolic excursion (TAPSE) of 10 mm to normal function with a TAPSE of 18 mm and LVEF of 33% (Figure 2B).

## Discussion

Our case presents a rare and noteworthy finding: the reversal of LBBB following alcohol cessation and initiation of guideline-directed medical therapy for HFrEF in a patient with alcoholic cardiomyopathy (ACM). This phenomenon is infrequently reported in the literature.

LBBB is a unique model of ventricular electromechanical desynchrony, and is suspected to generate structural remodeling, LV dysfunction, and worse prognosis.^1^ The prevalence of LBBB is <1%, but increases with age, up to 5% at 80 years.^1^ Most patients with LBBB have evidence of structural heart disease such as coronary artery disease, degenerative changes, infiltrative conditions, and cardiomyopathies, including ACM.^1^

Reversible bundle-branch block typically falls into seven categories: (1) rate-dependent ("functional") block that appears only at very fast or very slow heart rates; (2) transient ischemia or coronary spasm during acute myocardial infarction, resolving after reperfusion; (3) severe electrolyte disturbances, especially hyperkaliemia, in which normalization of serum K^+^ restores conduction; (4) sodium-channel–blocking drugs or toxins (e.g., flecainide, lacosamide, tricyclic antidepressants, digoxin intoxication); (5) inflammatory oedema from myocarditis of viral, autoimmune, Lyme, or checkpoint-inhibitor origin; (6) chest trauma or post-procedural septal oedema, which usually abates within 24–48h; and (7) acute hemodynamic overload or reversible cardiomyopathy in decompensated heart failure, where medical optimization and "reverse remodeling" can narrow the QRS.^2,3^

Although pharmacological treatment for heart failure is well known to induce reverse remodeling and improve clinical outcomes, reversal of LBBB is not commonly observed. In fact, studies suggest that in patients with heart failure and LBBB, cardiac resynchronization therapy (CRT) may be superior to medical therapy alone in improving cardiac function.^4^

Only a few cases in the literature describe reversible LBBB. A report suggested improvement of LBBB following treatment with sacubitril/valsartan, although the causality in that case is debatable.^5^ Notably, our patient experienced LBBB resolution without the use of sacubitril/valsartan, highlighting the potential contribution of other mechanisms, particularly alcohol cessation and optimized standard therapy for HFrEF.

Pathophysiologically, ACM is characterized by direct toxic effects of ethanol and its metabolites on the myocardium, promoting oxidative stress, mitochondrial dysfunction, inflammation, and fibrosis. These alterations not only impair myocardial contractility but can also affect the cardiac conduction system.^6^ Experimental studies in canine models have demonstrated that chronic alcohol exposure can lead to conduction delays and structural changes in the ventricular conduction system, supporting the hypothesis of potential reversibility with removal of the toxic insult.^7^

Furthermore, approximately one-half to two-thirds of patients with ACM have no myocardial fibrosis detectable by late gadolinium enhancement, a finding that likely reflects either an earlier disease stage or an individual susceptibility to functional injury without structural remodeling. The absence of fibrosis is linked to a better response to alcohol cessation and possibly to lower mortality.^6^

Proposed explanations for absent or minimal fibrosis include the exposure time and disease stage—myocardial dysfunction in ACM can emerge before extracellular-matrix deposition, especially in younger drinkers or those with shorter abuse duration; predominantly functional toxicity—metabolic (oxidative stress, mitochondrial dysfunction) and inflammatory changes may depress contractility without causing extensive necrosis; and modulating factors, such as zinc levels, polymorphisms in ethanol-metabolizing enzymes and cardiomyokines like FGF-21, which have been implicated in experimental models as protective against fibrogenesis.^8^

Thus, in our case, LBBB reversal may be explained by a combination of structural and functional myocardial recovery driven by both abstinence from alcohol and optimized medical therapy. This finding underscores the importance of alcohol cessation in the management of ACM and adds to the limited literature by documenting a rare case of electrocardiographic recovery in a clearly defined clinical setting.

Classic limitations of singularity and, therefore, limited generalizability include the absence of a control group or comparative series precludes establishing a firm causal link between the resolution of the left bundle-branch block and the combination of alcohol abstinence with pharmacologic uptitration; the short follow-up and paucity of serial ECGs or continuous monitoring do not allow the exact chronology to be defined or the durability of QRS normalization to be ensured; and multiple drugs were titrated concurrently, obscuring the isolated effect of each.

## Conclusion

In summary, this case illustrates an exceptionally rare reversal of LBBB in a patient with alcoholic cardiomyopathy, likely driven by optimized guideline-directed medical therapy and sustained alcohol cessation, leading to favorable cardiac remodeling.

## Data Availability

The underlying content of the research text is contained within the manuscript.
